# A Mutation of *lips-6* in *C. elegans*

**DOI:** 10.17912/micropub.biology.000419

**Published:** 2021-07-09

**Authors:** Sarah M Cohen, Henry H Le, Paul W Sternberg

**Affiliations:** 1 Division of Biology and Biological Engineering, California Institute of Technology, Pasadena, CA 91125, USA; 2 Boyce Thompson Institute and Department of Chemistry and Chemical Biology, Cornell University, Ithaca, New York 14853, United States

## Abstract

The *lips-6* gene encodes a putative lipase that plays a role in adult starvation response through a pathway that is parallel to the dauer pathway in larval *Caenorhabditis elegans *worms. We created a mutation of *lips-6* to study its effects on lifespan and the ascaroside profile. We found that *lips-6 *had a wild-type lifespan and a wild-type ascaroside profile. These results suggest that the *lips-6* gene plays a minimal role in *C. elegans* lifespan biology and does not affect the ascaroside profile until it is specifically activated by starvation in adult worms.

**Figure 1. lips-6 loss-of-function mutant phenotype:**
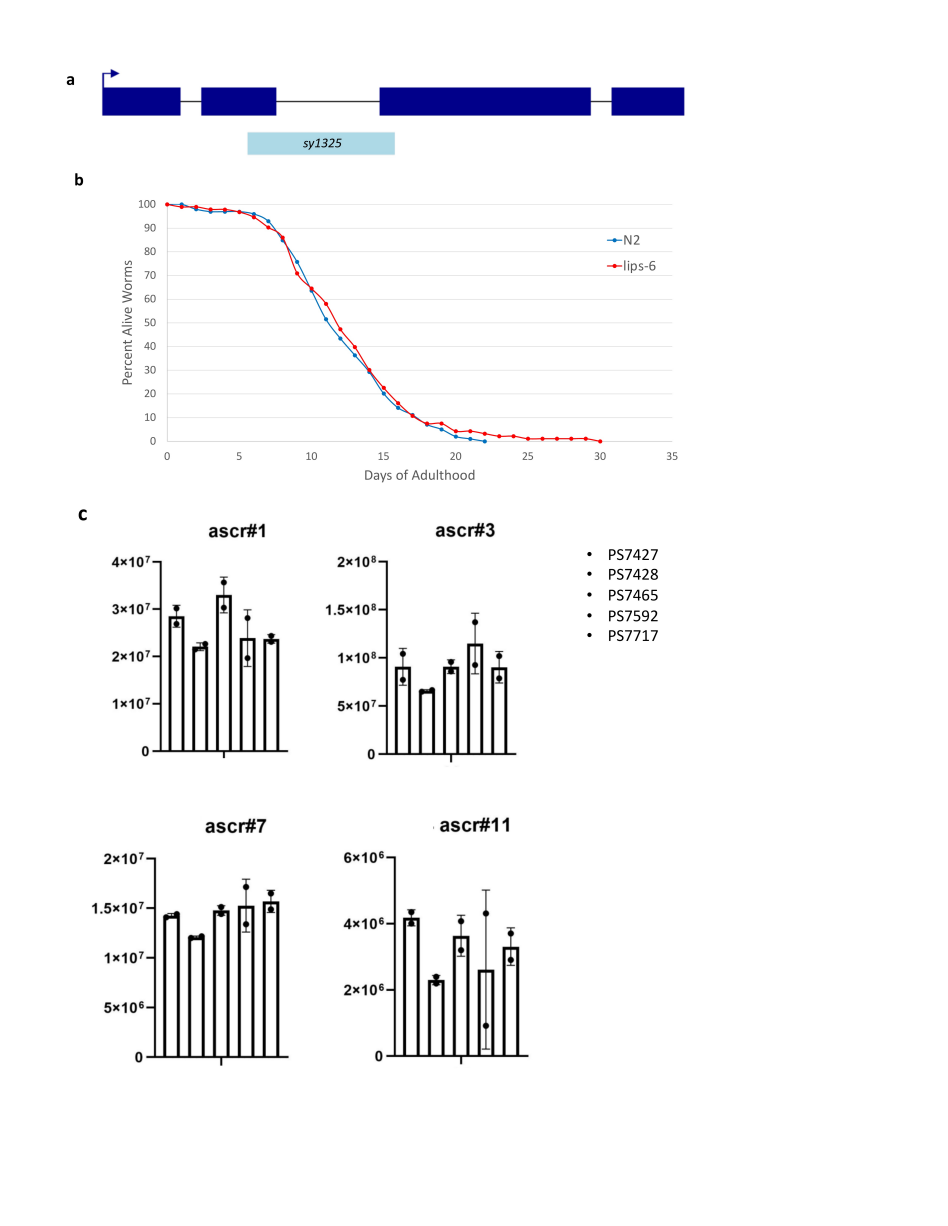
(a) We constructed a 348 basepair deletion *lips-6(sy1325)* mutant strain using CRISPR/Cas9. This deletion removes one-fourth of the gene. (b) Lifespan analysis of *lips-6* was wildtype. (c) Relative abundances of selected ascarosides of *lips-6(sy1325),* indicated here by its strain number PS7427 (first column), as compared to several unrelated strains exemplifies that there are no major changes in the ascaroside profile of *lips-6.*

## Description

The efficient CRISPR/Cas9 genome editing toolkit in the nematode *Caenorhabditis elegans* has made the creation of null mutants easier than ever.The putative lipid hydrolase *lips-6* is known to function in the DAF-12/DIN-1 pathway to promote fat mobilization during periods of starvation in adult worms (Tao *et al.* 2016). Similarly, it was found to be strongly downregulated in response to dauer ascarosides given during the L1 to L2d decision in larval worms (Cohen *et al.* 2021). Here, we have created a mutant of the putative lipase *lips-6* and studied its lifespan as well as its ascaroside profile.

First, we made a mutation of *lips-6* that has a ­­­348 base pair deletion, spanning one-quarter of the entire gene (Figure 1a). We performed a longevity assay on *lips-6(sy1325)* and found that it has a wild-type lifespan phenotype (Figure 1b). We also tested the ascaroside profile of *lips-6(sy1325)* and found that, when compared to a series of unrelated strains, the ascaroside profile was also wild-type. Our results indicate that until the *lips-6* gene is activated by starvation during adulthood, it plays no role in lifespan or the worms’ changing ascaroside profile.

## Methods

**Strain Creation:** We used the CRISPR/Cas9 method essentially as described in Köhler *et al.* (2017). We used the guide sequence AATACTTATTCGCAACAACA. To detect a deletion mutation of *lips-6*, we used forward primer ­­­­TACGGTAGCCGTTATGCTTG and reverse primer GCTGATGTGACTGCTGTTCG. In *sy1325*, there is an in-frame deletion of 348 base pairs traversing one-quarter of the gene; the deletion flanking sequences are ACGACAATACTTATTCGCAA_deletion_GGAGCTCAGAAAATTGACGT.

**Longevity Assays:** The longevity assays were done using the method described in Larsen *et al.* (1995). Ten adult worms were kept on each 6cm plate in order to prevent food scarcity or overcrowding – 100 worms were assayed for each strain. The first day of the survival curve is the first day of adulthood. Worms were scored every day as alive, dead, or missing. Worms were deemed dead if they neither moved, pumped, nor responded to stimulation using the end of a platinum wire pick.

**Nematode Cultures:** Cultures were started by picking 20 *C. elegans* hermaphrodites onto 10cm NGM agar platescontaining OP50 *E. coli* and incubated at 22°C. After 96 h, each plate was washed with 25 mL of S-complete medium into a 125 mL Erlenmeyer flask, 1 mL of concentrated *E. coli* OP50 was added (see above), and cultures were shaken at 220 RPM at 22°C. After 72 h, cultures were centrifuged at 1000 G for 1 min. After discarding the supernatant, worm pellets were washed with 30 mL of ddH2O and centrifuged at 1,000 G for 1 min, and the supernatant was discarded. 24 mL ddH2O was added, along with 6 mL regular bleach (Clorox, Oakland, California) and 900 uL 10 M NaOH, and the mixture was shaken for 2.5 min to prepare eggs. Eggs were centrifuged at 1,000 G and the supernatant was removed. Eggs were then washed with 30 mL M9 buffer and suspended in a final volume of 5 mL M9 buffer in a 50 mL centrifuge tube. Eggs were placed on a rocker and allowed to hatch for 24 h at 22°C. L1 larvae were counted and seeded at 70,000 L1s per 25 mL S-complete culture, and 1 mL of OP50 was added per 25 mL culture in 125 mL Erlenmeyer flasks. Cultures were incubated at 220 RPM at 22°C for 72 h.

**Metabolite Extraction:** Lyophilized culture media were crushed and homogenized by shaking with 2.5 mm steel balls at 1300 RPM for 3 min in 30 s pulses while chilled with liquid nitrogen (SPEX sample prep miniG 1600). Dried material was then extracted with 15 mL methanol in 50 mL centrifuge tubes, rocking overnight at 22°C. Extractions were pelleted at 5000 G for 10 min at 4°C, and supernatants were transferred to 20 mL glass scintillation vials. Samples were then dried in a SpeedVac (Thermo Fisher Scientific) vacuum concentrator. Dried materials were resuspended in 1 mL methanol and vortexed for 1 min. Samples were pelleted at 5000 G for 5 min and 22°C, and supernatants were transferred to 2 mL HPLC vials and dried in a SpeedVac vacuum concentrator. Samples were then resuspended in 200 uL of methanol, transferred into 1.7 mL Eppendorf tubes, and centrifuged at 18,000 G for 20 min at 4°C. Clarified extracts were transferred to fresh HPLC vials and stored at −20°C until analysis.

**Mass Spectrometric Analysis:** High resolution LC−MS analysis was performed on a ThermoFisher Scientific Vanquish Horizon UHPLC System coupled with a Thermo Q Exactive HF hybrid quadrupole-orbitrap high resolution mass spectrometer equipped with a HESI ion source. Metabolites were separated using a water-acetonitrile gradient on an Agilent Zorbax Eclipse XDB-C18 column (150 mm × 7 2.1 mm, particle size 1.8 um) maintained at 40°C. Solvent A: 0.1% formic acid in water; Solvent B: 0.1% formic acid in acetonitrile. A/B gradient started at 1% B for 5 min after injection and increased linearly to 100% B at 20 min, using a flow rate 0.5 mL/min. Mass spectrometer parameters: spray voltage 3.0 kV, capillary temperature 380°C, probe heater temperature 300°C; sheath, auxiliary, and spare gas 60, 20, and 2, respectively; S-lens RF level 50, resolution 240,000 at m/z 200, AGC target 3×106. The instrument was calibrated with negative ion calibration solutions (ThermoFisher).

## Reagents

All CRISPR/Cas9 system reagents were ordered from IDT except for the Cas9 protein, which was kindly provided by Tsui-Fen Chou. Sequences were downloaded from WormBase.

Strain Generated:

PS7427 *lips-6(sy1325)* IV

List of Strains.

**Table:** 

**Gene**	**Strain**	**Source**
*C. elegans* Wildtype	N2	Brenner, 1974; Caenorhabditis Genetics Center (CGC)
*lips-6(sy1325)*	PS7427	This work
*oac-39(sy1321)*	PS7428	Sternberg Lab Collection
*oac-54(sy1322)*	PS7465	Sternberg Lab Collection
*oac-9(sy1324)*	PS7592	Sternberg Lab Collection
*oac-55(sy1323)*	PS7717	Sternberg Lab Collection
